# The herbal formula KH-204 is protective against erectile dysfunction by minimizing oxidative stress and improving lipid profiles in a rat model of erectile dysfunction induced by hypercholesterolaemia

**DOI:** 10.1186/s12906-017-1588-4

**Published:** 2017-02-24

**Authors:** Hoon Jang, Woong Jin Bae, Su Jin Kim, Hyuk Jin Cho, Seung Mo Yuk, Dong Seok Han, Chang Shik Youn, Eun Bi Kwon, Sung Yeoun Hwang, Sae Woong Kim

**Affiliations:** 1 Department of Urology, College of Medicine, The Catholic University of Korea, 222 Banpo-daero, Seocho-gu, Seoul 137-701 Republic of Korea; 2 Catholic Integrative Medicine Research Institute, College of Medicine, The Catholic University of Korea, Seoul, South Korea; 3 KEMIMEDI, Seoul, South Korea; 4 Department of Urology, College of Medicine, The Catholic University of Korea, 222 Banpo-daero, Seocho-gu, Seoul 06591 South Korea

**Keywords:** Erectile dysfunction, Hypercholesterolaemia, Oxidative stress

## Abstract

**Background:**

Hypercholesterolaemia (HC) is a major risk factor for ischemic heart disease and is also known to be a risk factor for erectile dysfunction (ED). ED caused by HC is thought to be related to HC-induced oxidative stress damage in the vascular endothelium and erectile tissue. KH-204 is an herbal formula with a strong antioxidant effect. We evaluated the effects of KH-204 on erectile function in a rat model of HC-induced ED.

**Methods:**

Male Sprague-Dawley rats (6 weeks old) were divided into normal control, high-fat and cholesterol diet (HFC), and HFC with KH-204 treatment (HFC + KH) groups (*n* = 12 each). Normal control group rats were fed normal chow diet. HFC and HFC + KH group rats were fed high-fat and cholesterol diets and treated with or without daily oral doses of KH-204 for 12 weeks. Subsequently, intracavernous pressure (ICP) and mean arterial pressure (MAP) were measured, and lipid profiles, expression of endothelial (eNOS) and neuronal (nNOS) nitric oxide synthase, oxidative stress (8-hydroxy-2-deoxyguanosine), and ratio of smooth muscle cells and collagen fibres were evaluated in the serum and corpora tissue.

**Results:**

Compared to the HFC group, the HFC + KH group showed statistically significant increases in peak ICP and ICP/MAP ratio, expression of eNOS and nNOS, and ratio of smooth muscle cells and collagen fibres (*p* < 0.05). The HFC + KH group also showed statistically significant decreases in oxidative stress (*p* < 0.05). Further the lipid profiles of this group were ameliorated compared to those of the HFC group (*p* < 0.05).

**Conclusions:**

The current study shows that the antioxidant and hypolipidemic effects of KH-204 are effective in ameliorating ED by restoring endothelial dysfunction and suggests that KH-204 may be a potential therapeutic agent for ED by correcting the fundamental cause of ED.

## Background

Penile erection is a complex physiological process that occurs through a cascade of neurogenic, vascular, and humoral events. To maintain a penile erection for satisfactory sexual intercourse, important preconditions are necessary, including proper cavernosal innervation, maintenance of endothelial function, and well-functioning erectile tissue. Erectile dysfunction (ED), defined as the consistent inability to attain or maintain a penile erection of sufficient quality to permit satisfactory sexual intercourse [1], is a highly prevalent global health problem with considerable impact on the quality of life of middle-aged men [2]. ED can be induced by various causes, including arterial, neurogenic, hormonal, cavernosal, iatrogenic, and psychogenic causes. However, the most common cause is likely related to vascular abnormalities caused by endothelial dysfunction and erectile tissue dysfunction [3, 4].

Hypercholesterolaemia (HC) is a metabolic disorder characterized by elevated levels of total cholesterol (TC) in the blood. HC may develop as a consequence of an unbalanced diet, obesity, inherited (genetic) disease (familial hypercholesterolaemia), or another disease (e.g. diabetes). Approximately 30.9 million people (13.1%) were reported to have HC in the United States in 2012 [5].

It is well known that HC is a major risk factor for the development of atherosclerosis and subsequent ischemic heart disease [6]. Elevated serum cholesterol and reduced high-density lipoprotein cholesterol (HDL-C) levels are also associated with an increased risk of ED [7]. The relationship between ED and HC has been demonstrated by several studies [8, 9]. Although the mechanism underlying the increased ED risk by HC has not been clearly elucidated, many studies reported an association between HC-induced oxidative stress damage in vascular endothelium and erectile tissue and ED [10–12].

KH-204 is an herbal formula composed of *Cornus officinalis* Sieb. et Zucc, *Lycium chinense* Miller, *Rubus coreanus* Miquel, *Cuscuta chinensis* Lam, and *Schisandra chinensis* Baillon. Several studies have reported that each herb or various combinations of these herbs have a number of beneficial properties, including antioxidant [13–18], anti-inflammatory [15, 19], anti-apoptotic [17, 20, 21], anti-fibrotic [15, 22, 23], and hypolipidemic effects [24, 25] in multiple diseases. These herbs have been widely used in Korea as medicines for many years. Ojayounjonghwon, an herbal formula described in Dong Ui Bo Gam (a representative of Korean traditional medicine books), includes four of the above herbs, with the exception of *Cornus officinalis* Sieb. et Zucc., and has been used for the treatment of late onset hypogonadism (LOH) symptoms, including ED. KH-204 is a new herbal formula that is a modified form of Ojayounjonghwon. Park et al. reported that KH-204 improved erectile function in an aged and diabetic rat model of ED by restoring or activating the nitric oxide (NO)-cyclic guanosine monophosphate (cGMP) pathway and synergistically activating nitric oxide synthase (NOS) [26]. In addition, Sohn et al. reported that KH-204 improved erectile function in a spontaneous hypertensive rat model of ED by increasing the expression of endothelial-NOS (eNOS) and neuronal-NOS (nNOS) [27]. We hypothesized the improvement of erectile function by KH-204 treatment was attributable to its antioxidant effects.

The aim of the present study was to assess the effects of HC on the quality of erections and to evaluate the effects of KH-204 treatment in a rat model of ED induced by HC.

## Methods

### Preparation of KH-204

The major ingredients of KH-204 are fruits obtained from five plants: *Cornus officinalis* Sieb. et Zucc. (CO; 32%), *Lycium chinense* Miller (LC; 32%), *Rubus coreanus* Miquel (RC; 16%), *Cuscuta chinensis Lam* (CC; 16%), and *Schisandra chinensis* Baillon (SC; 4%). These herbs were purchased from Andong Excellent Medicinal Herbs Distribution Center Co., Ltd. (Andong, Korea), and identified by one of the authors (S.Y. Hwang). Voucher specimens (KH204-CO, KH204-LC, KH204-RC, KH204-CC, and KH204-SC) of each plant were deposited at the R&D centre of KEMIMEDI (KMD) Co. Ltd. (Andong, Korea). Each herb (20 kg) was extracted in 200 L of distilled 30% ethanol and refluxed at 98 ± 2 °C for 3 h. The extract was filtered, and the liquid from the filtrates was removed by a rotary evaporator and a spray dryer. KEMIMEDI (KMD) Co. Ltd. (Seoul, Korea), a venture company that develops Oriental herbal medicines, developed this product as a health supplement.

### Marker Compounds for Each Plant

A marker compound for each plant was selected, and their chemical structures are shown in Table 1.

**Table 1:** Marker compound and chemical structure in each plant

Plant source	Marker compound	Chemical structure
*Cornus officinalis* Sieb. et Zucc *(KH204-CO)*	Loganin	
*Lycium chinense* Miller *(KH204-LC)*	Betain	
*Rubus coreanus* Miquel *(KH204-RC)*	Ellagic acid	
*Cuscuta chinensis* Lam *(KH204-CC)*	Hyperoside	
*Schisandra chinensis* Baillon *(KH204-SC)*	Schisandrin	

**Fig. 1:**
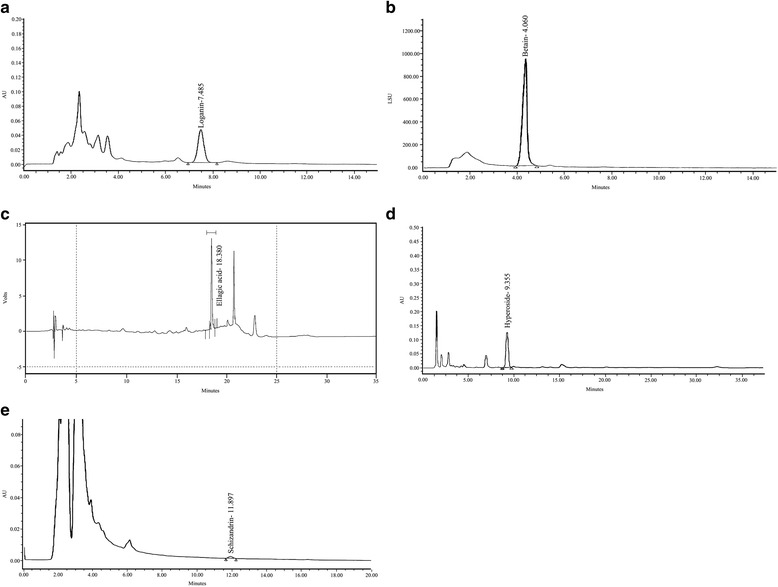
HPLC chromatogram of each plant. **a** Loganin is the marker compound of *Cornus officinalis* Sieb. et Zucc. **b** Betain is the marker compound of *Lycium chinense* Miller. **c** Ellagic acid is the marker compound of *Rubus coreanus* Miquel. **d** Hyperoside is the marker compound of *Cuscuta chinensis* Lam. **e** Schizandrin is the marker compound of *Schisandra chinensis* Baillon

The presence of the marker compound for each plant was confirmed by high-performance liquid chromatography (HPLC). Each peak in the HPLC profile was identified by comparison with the retention times and UV spectra of standard compounds (Fig. 1).

### Animal Groups and Treatment Protocol

Thirty-six 6-week-old male Sprague-Dawley rats, supplied by Orient Bio Inc. (Gyeonggi-do, Korea), were treated under a protocol approved by the Institutional Animal Care and Use Committee at the School of Medicine, The Catholic University of Korea (Approval Number: CUMC-2016-0111-01) and handled according to the guidelines of the National Institutes of Health (NIH). Rats were divided equally into three groups (*n* = 12 each): Control, the normal control group; HFC, the HC group induced by high fat and cholesterol diet; and HFC + KH, HFC rats administered 400 mg/kg of KH-204. Animals were housed three per plastic cage and identified by the presence or absence of ear punches. The lighting of the animal rooms was controlled by artificial light for 12 h from 7 o’clock in the morning to 7 o’clock in the evening, and 18–23 °C room temperature and 40–60% humidity were maintained. All rats in the HFC and HFC + KH groups received a high-fat and cholesterol diet, supplied by Feedlab, (Gyionggi-do, Korea). Rats in the Control group received a normal chow diet for 12 weeks. The formulation of the HFC diet is shown in Table 2. Rats in the HFC + KH group received KH-204 (400 mg/kg) for 12 weeks. KH-204 was dissolved in distilled water and administered orally through an 8 F red Rob-Nel catheter once a day. After 12 weeks, all rats were weighed and underwent intracavernosal pressure (ICP) measurement. After ICP measurement, blood from the internal carotid artery and corporal tissue were sampled.

**Table 2:** Composition of the high fat and cholesterol diet

Formulation	High Fat and Cholesterol Diet (HFD 45% cal + 2% Cholesterol)
	(gm%)
Protein	24
Carbohydrate	41
Fat	24
Ingredient	g
Casein (from milk)	233.1
Corn Starch	84.8
Sucrose	201.4
Dextrose	116.5
Cellulose	58.3
Soybean Oil	29.1
Lard	206.9
Mineral mixture	52.4
Vitamin mixture	11.7
TBHQ	0.0
L-Cysteine	3.5
Choline bitartrate	2.3
Total	1,000.0
Cholesterol	20
Cholic acid	5

*HFD* high-fat diet, *TBHQ* tertiary-butylhydroquinone

### Serum Level of Total Cholesterol (TC), Low Density Lipoprotein (LDL)/Very Low Density Lipoprotein (VLDL) Cholesterol, High Density Lipoprotein (HDL) Cholesterol, and Triglyceride

To measure serum TC, LDL/VLDL-C, HDL-C, and TG, commercial assay kits [HDL and LDL/VLDL Cholesterol Assay Kit, STA-391 (Cell Biolabs Inc., San Diego, CA, USA); Triglyceride Quantification Colorimetric/Fluorometric Kit, K622-100, Biovision Inc. (Milpitas, CA, USA)] were used according to manufacturer instructions. Absorbance at 570 nm was read with a PowerWave HT Microplate Spectrophotometer (BioTek Instruments Inc., Winooski, VT, USA). Sample cholesterol concentrations were determined by interpolation from a standard curve.

### ICP Measurement

Rats were anaesthetised with an intraperitoneal injection of 0.2 ml tiletamine (Zoletil®). With the rat in the supine position, the penis was dissected, and the corpus cavernosum and crus of the penis were exposed. A low, midline abdominal incision was made to access the pelvis, and the pelvic ganglion lateral to the right prostate was exposed. For the measurement of ICP, a heparinised 23-gauge butterfly needle was inserted in the corpus cavernosum of penile proximal portion after the penile skin was degloved and the corpus cavernosum identified. Then, a bipolar electrical stimulator was placed on the ganglion to stimulate the cavernosal nerve for 50 s at 10 V and 2.4 mA for 0.5 ms. Cavernosal nerve stimulation was conducted at least three times and the interval between stimulations was maintained for more than 10 min. Both mean arterial pressure (MAP) and ICP were continuously monitored during electrical stimulation. Systemic MAP was continuously monitored via a 24-gauge polyethyl tube placed in the right carotid artery. Each 23-gauge butterfly needle was inserted in the right and left penile crus for recording ICP. Both the polyethyl tube and the butterfly needle were connected to tubing diluted with heparin (250 IU/ml). ICP and MAP were recorded via pressure transducers connected to a recorder (Grass model S48K, Grass Instrument Division, Astro-Med, West Warwick, RI, USA). Comparisons were made for ICP/MAP and area under the curve corresponding to the duration of electrical stimulation [28]. After the stimulation test, the corpus cavernosum was removed and divided in two. The first part was cryopreserved in liquid nitrogen, and the other part was fixed in formalin.

### Masson’s Trichrome Staining

Masson Trichrome staining was performed in paraffin-embedded corporal tissue sections. After ICP, the skin-denuded middle portion of the penile shafts were fixed overnight in 10% formalin, washed, and stored in 70% alcohol at 4 °C until they were processed for paraffin-embedded tissue sectioning (4 μm). After staining, the colour distribution of the muscle tissue was approximated using Adobe Photoshop CS 8.0. The entire colour distribution of the image was calculated and the muscle tissue distribution was selected and expressed as red in colour [28]. There was variation in our calculations due to colour overlays and ambiguity of the colour spectrum in the muscle tissues.

### eNOS Expression Test: Western Blot Analysis

Corpora tissue was homogenised in ice-cold lysis buffer containing 20 mM Tris-Cl, pH 8.0, 150 mM NaCl, 1 mM EDTA, 1% Triton X-100, 10 μM leupeptin, 20 μg/ml chymostatin, and 2 mM phenylmethanesulphonyl fluoride (PMSF). Following centrifugation at 12,000 *g* for 20 min at 4 °C, the supernatant was extracted and quantified using the bicinchoninic acid (BCA) protein assay kit (Pierce, Rockford, IL, USA). Proteins were separated by sodium dodecyl sulphate polyacrylamide gel electrophoresis (SDS-PAGE) and electrophoretically transferred to membranes. The membranes were blocked in 5% non-fat milk in Tris-buffered saline containing 0.1% Tween 20 and then probed with anti-eNOS antibody (1:1000; ab5589, Abcam, Cambridge, UK), anti-phosphorylated-eNOS (P-eNOS) antibody (1:1000; #9571, Cell Signaling Technology, Beverly, MA, USA), and anti-β-actin antibody (1:10000; SC47778, Santa Cruz Biotechnology, Dallas, TX, USA) as an internal control. After washing, membranes were incubated with horseradish peroxidase-conjugated secondary antibodies (Santa Cruz Biotechnology). Densitometric analysis of band intensity was detected by Luminescent Image Analysis System (LAS-3000; Fujifilm, Tokyo, Japan) and measured using Multi Gauge 3.0 software (Fujifilm) [29].

### nNOS Expression Test: Immunohistochemistry

The paraffin sections of corpora tissue were immunostained with the following primary antibodies: neuron-specific β-III tubulin diluted 1:200 (Abcam) and nNOS diluted 1:200 (Santa Cruz Biotechnologies). Sections were mounted with 4,6-diamidino-2-phenylindole (DAPI; Vector laboratories, Inc., Burlingame, CA, USA) to stain the nuclei. Digital images were obtained using a Zeiss LSM 510 Meta confocal microscope (Zeiss, Oberkochen, Germany), and the mean intensity was calculated using Zen 2009 (Zeiss) [30].

### Measurement of 8-Hydroxy-2-deoxyguanosine (8-OHdG) in Corpora Tissue

Oxidative stress in corpora tissues was evaluated by measuring the levels of 8-OHdG, oxidatively modified DNA. The DNeasy Blood and Tissue kit (Qiagen, Valencia, CA, USA) and the Total DNA oxidation kit (Highly Sensitive 8-OHdG Check enzyme linked immunosorbent assay (ELISA); Japan Institute for the Control of Aging, Fukuroi, Japan) were used according to the manufacturer’s protocol. The 8-OHdG standard (0.5–40 ng/ml) or 15–20 μg of DNA purified from the testis was incubated for 1 h with a monoclonal antibody against 8-OHdG in a microtiter plate precoated with 8-OHdG. After development of the final colour with the addition of 3,3′,5,5′-tetramethylbenzidine, absorbance was measured at 450 nm. Tissue sample concentration was calculated from a standard curve and was corrected for DNA concentration [28].

### Statistical Analysis

All data are presented as the mean ± standard deviation (SD). Data were analysed using SPSS 15.0 (SPSS Inc., Chicago, IL, USA). Data were evaluated using analysis of variance (ANOVA), with group comparisons made by Scheff’s test. A *p*-value less than 0.05 was considered to be statistically significant.

## Results

### Baseline Data Characteristics

The test results of four (one in the control, two in the HFC, and one in the HFC + KH group) of the total 36 rats were excluded from the baseline data because of anaesthesia-related deaths and/or a failure to catheterize the internal carotid artery.

### Body Weight Gain and Serum Lipid Profiles

Body weight gain and serum levels of TC, LDL/VLDL-C, HDL-C, and TG of the three groups are shown in Table 3. Over the course of the experiment, body weight gain was significantly higher in the HFC group than in the control group. Compared with the HFC group, administration of KH-204 significantly reduced body weight gain. Regarding cholesterol and TG levels, serum TC, LDL/VLDL-C and TG levels increased and HDL-C levels decreased in the HFC group compared with the control group, whereas the lipid profiles in the KH-204 treatment group were significantly improved compared to the HFC group.

**Table 3:** Body weight gain, lipid profiles, and oxidative stress in each group

	Control	HFC	HFC + KH	*p* value
Body Weight Gain (%)	222.57 ± 17.81	255.40 ± 25.94	236.17 ± 20.15	0.008^a^ 0.007^b^ 0.048^c^
Lipid Profiles				
Total Cholesterol (μM)	91.71 ± 6.68	168.77 ± 26.66	127.83 ± 8.83	<0.001^a^ <0.001^b^ <0.001^c^
LDL/VLDL-Cholesterol (μM)	47.65 ± 5.45	134.66 ± 27.28	87.27 ± 7.54	<0.001^a^ <0.001^b^ <0.001^c^
HDL-Cholesterol (μM)	44.06 ± 2.40	34.12 ± 2.83	40.56 ± 2.83	<0.001^a^ <0.001^b^ <0.001^c^
Triglyceride (mM)	1.14 ± 0.16	2.76 ± 0.19	1.76 ± 0.27	<0.001^a^ <0.001^b^ <0.001^c^
8-OHdG (ng/mL)	2.88 ± 1.03	10.57 ± 1.42	6.06 ± 1.16	<0.001^a^ <0.001^b^ <0.001^c^

Data are expressed as mean ± standard deviation. *HFC* high fat and cholesterol diet, *KH* KH-204, *LDL* low density lipoprotein, *VLDL* very low density lipoprotein, *HDL* high density lipoprotein, *8-OHdG* 8-Hydroxy-2-deoxyguanosine

^a^One-way ANOVA test, overall comparison

^b^Comparison between the control and HFC groups

^c^Comparison between the HFC and HFC + KH groups

### In vivo Assessment of Erectile Function

Peak ICP and ICP/MAP ratios decreased in the HFC group compared to the control group and significantly improved in the HFC + KH group. The control and HFC + KH groups had statistically similar peak ICP (*p* = 0.02) and ICP/MAP ratios (*p* = 0.13), which were significantly higher than those of the HFC group (Table 4).

**Table 4:** Intracavernous pressure (ICP) in response to electrical stimulation of the cavernous nerve in rats from each experimental group

	Control	HFC	HFC + KH	*p* value
Peak ICP	105.86 ± 10.38	28.29 ± 8.69	78.75 ± 30.56	<0.001^a^ <0.001^b^ <0.001^c^
ICP/MAP ratio	0.83 ± 0.12	0.27 ± 0.05	0.70 ± 0.25	<0.001^a^ <0.001^b^ <0.001^c^

Data are expressed as mean ± standard deviation

^a^One-way ANOVA test, overall comparison

^b^Comparison between the control and HFC groups

^c^Comparison between the HFC and HFC + KH groups

### Masson’s Trichrome Staining

**Fig. 2:**
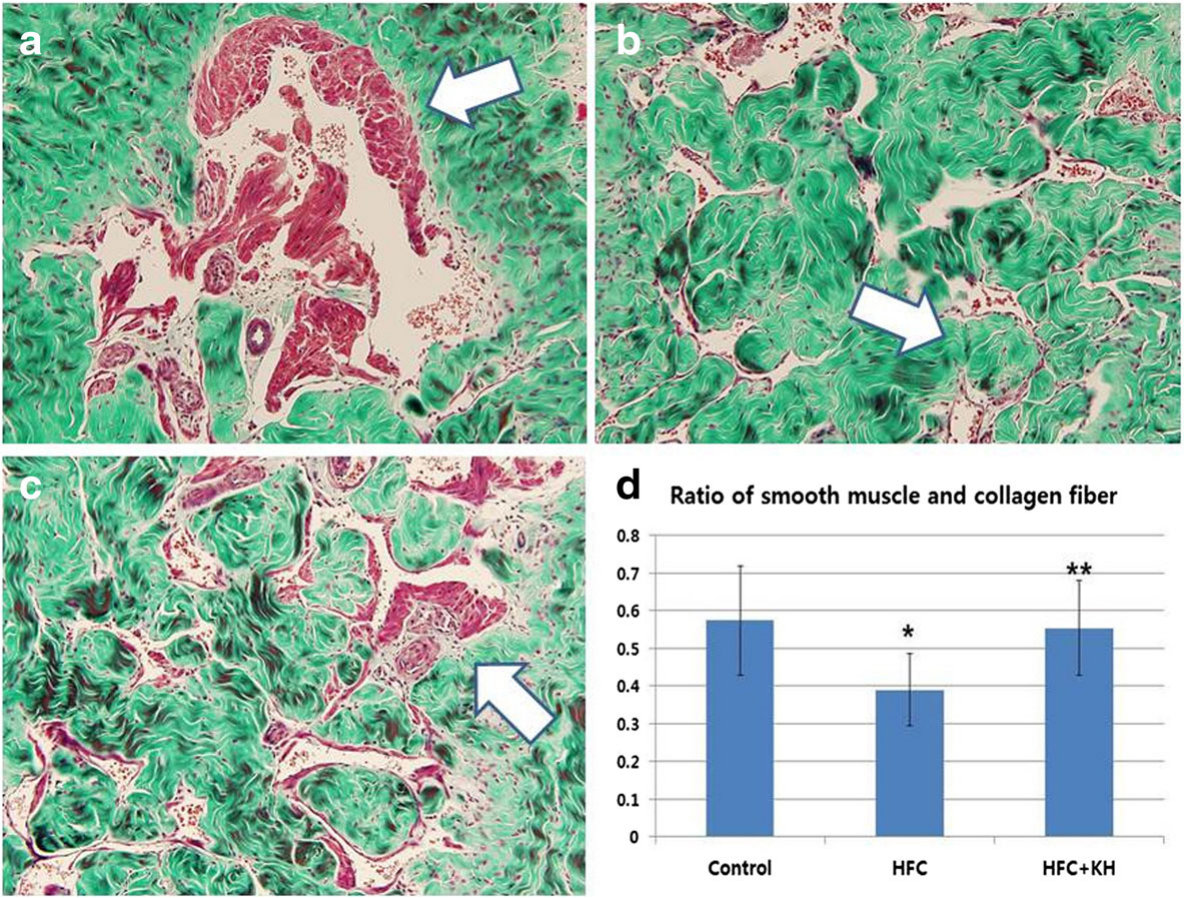
Masson’s trichrome staining of corpora tissue. **a**, **b**, **c** Masson’s trichrome staining of corpora tissue: smooth muscle is shown in red (white arrow) and collagen is shown in green (Magnification: x200). **d** Ratio of smooth muscle and collagen fibres in corpora tissue. Data are expressed as mean ± standard deviation (SD). * = Significant difference between the control and high fat and cholesterol diet (HFC) groups. ** = Significant difference between the HFC and HFC + KH groups

Compared to the control group, the HFC group exhibited decreased corpora tissue smooth muscle content and increased collagen deposition (Fig. 2a and b). In the HFC + KH group, however, smooth muscle content increased and collagen content decreased compared to the HFC group, and the levels of these parameters were similar to those in the control group (Fig. 2a and c). The mean muscle/collagen ratio ± SD for the control, HFC, and HFC + KH groups were 0.57 ± 0.15, 0.39 ± 0.10, and 0.55 ± 0.13 respectively (Fig. 2d). The muscle/collagen ratio significantly decreased in the HFC group compared to the control group, and KH-204 treatment significantly improved the muscle/collagen ratio in HFC rats.

### Expression of eNOS in Corpora Tissue

**Fig. 3:**
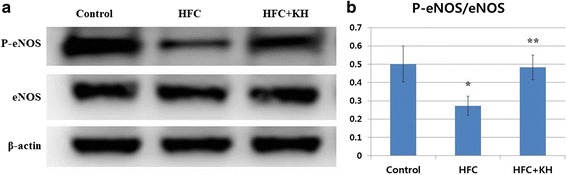
Phosphorylated-endothelial nitric oxide synthase (eNOS) protein expression in corpora tissue. **a** Western blot analysis of P-eNOS and eNOS in corporal tissue. **b** Densitometric analysis of P-eNOS relative to eNOS. Data are expressed as mean ± SD. * = Significant difference between the control and high fat and high cholesterol diet (HFC) groups. ** = Significant difference between the HFC and HFC + KH groups

We examined the protein expression of eNOS and P-eNOS in the corpora tissue by Western blot (Fig. 3a). There were no differences in total eNOS protein expression observed among the three groups. However, P-eNOS protein expression was significantly lower in the HFC group compared to the control group (*p* < 0.01), and this decrease was significantly attenuated in the HFC + KH group. The protein expression of P-eNOS was statistically nonsignificant between the control and HFC + KH groups (*p* = 0.23), with both groups being significantly higher than the HFC group. The mean densities ± SD of P-eNOS/eNOS for the control, HFC, and HFC + KH groups were 0.50 ± 0.10, 0.27 ± 0.05, and 0.48 ± 0.07, respectively (Fig. 3b).

### Expression of nNOS in Corpora Tissue

**Fig. 4:**
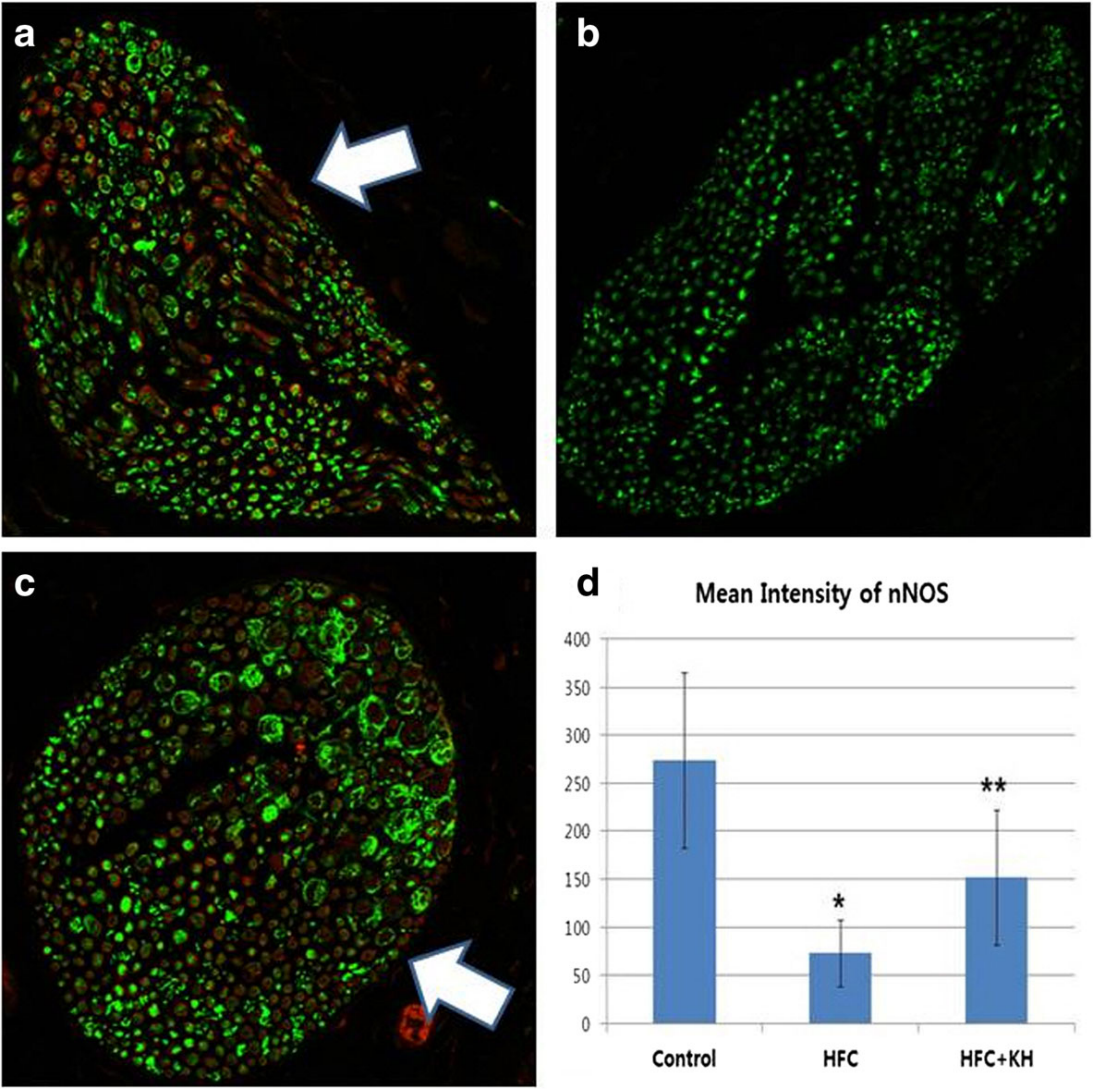
Immunostaining of neuronal nitric oxide synthase (nNOS) in the dorsal penile nerve. **a**, **b**, **c** Immunostaining for nNOS (red - white arrow) and β-III tubulin (green) in the dorsal penile nerve. Magnification: x400. **d** Mean intensity of nNOS expression for the dorsal penile nerve cross section. Data are expressed as mean ± SD. * Significant difference between the control and high fat and cholesterol diet (HFC) groups. ** Significant difference between the HFC and HFC + KH groups

In the dorsal penile nerve, expression of nNOS was analysed by immunohistochemical staining (Fig. 4a, b and c). The mean intensities ± SD of nNOS-positive areas for the control, HFC, and HFC + KH groups were 267.44 ± 98.61, 67.18 ± 40.38, and 151.5 ± 70.65, respectively (Fig. 4d). The expression of nNOS significantly decreased in the HFC group compared with the control group (*p* < 0.01), whereas its expression was significantly greater in the HFC + KH group than in the HFC group (*p* = 0.01) (Fig. 4d).

### Measurement of Oxidative Stress in Corpora Tissues

Oxidative stress levels in corpora tissues were assessed quantitatively by measuring 8-OHdG in the corpora tissue using ELISA. Oxidative stress was significantly greater in the HFC group than in the control group (*p* < 0.01). In the HFC + KH group, however, oxidative stress significantly decreased compared to the HFC group (*p* < 0.01) (Table 3).

## Discussion

The main findings of the present study were as follows: (1) treatment with KH-204 restored erectile function by improving lipid profiles and decreasing oxidative stress in a rat model of ED induced by HC; (2) treatment with KH-204 activated the protein expression of eNOS and nNOS, leading to an increase in NO bioactivity and an improvement in erectile function; and (3) KH-204 treatment decreased 8-OHdG levels, confirming the antioxidant effect of KH-204. Furthermore, the increase in the expression of eNOS, nNOS, and the muscle/collagen ratio with KH-204 treatment is consistent with the antioxidant effect of KH-204.

Although the precise mechanism underlying HC-associated ED has not been clearly elucidated, several studies have reported distinguishing findings in men and animals with HC. These findings included impairment of endothelium-dependent and endothelium-independent relaxations of the corpus cavernosum, decreased cavernosal endothelial cell content, altered smooth muscle cell function, and increased collagen content [10, 31, 32]. These results are thought to be related to oxidative stress damage by HC in the vascular endothelium and erectile tissue [10–12].

HC has been shown to increase the protein expression of nicotinamide adenine dinucleotide phosphate (NAD(P)H) oxidase and to induce eNOS uncoupling in the penis [33]. Activated NAD(P)H oxidase and eNOS uncoupling are known to lead to the formation of vascular superoxide anions, which induces an imbalance between the formation of superoxide anions and the detoxification of superoxide anions by antioxidant defence systems. In addition, superoxide anions may directly inactivate NO and decrease its bioavailability. NO is the most important neurotransmitter mediating the relaxation of the smooth muscle layer present in the corpus cavernosum [13, 19]. NO is an endothelium-derived relaxation factor and plays an important role in the regulation of the bioavailability of NO. NOS exists in three isoforms: nNOS, eNOS, and inducible NOS. NO is produced by eNOS in the endothelium of the corpus cavernosum and penile artery, and it is secreted form nonadrenergic and noncholinergic nerve endings [20]. NO and superoxide anions can react to form reactive nitrogen species, such as the highly toxic molecule peroxynitrite. Peroxynitirite may cause oxidative damage to DNA, proteins, and lipids, as well as eNOS uncoupling, release of vasoconstrictors, apoptosis, tissue injury, and inflammation [34]. Therefore, oxidative stress promoted by an increase in superoxide anions may impair the expression of eNOS and nNOS [14, 15, 22, 23], reduce eNOS activity and NO production [15–17, 22, 23, 35, 36], increase caveolin-1 expression [21] and its interaction with eNOS [11], and dysregulate cGMP signal transduction pathways [12]. Taken together, these findings suggest that ED occurs in HC.

In our study, the rats in the HFC group showed decreased expression of eNOS and nNOS, increased level of 8-OHdG, and decreased muscle/collagen ratio in the corpus cavernosum compared to the control group. These findings suggest that HC due to a high fat and cholesterol diet induced oxidative stress, leading to vascular endothelial and corporal tissue damage. This damage was detected as decreases in the ICP and ICP/MAP ratio compared to control.

The beneficial effects of KH-204, which are due to its antioxidant properties, have been reported in several studies. Bae, et al. found that KH-204 treatment decreased the expression of Rho kinase and increased the expression of cGMP in androgen-deprived rat bladder and that KH-204 may prevent deleterious molecular changes in the bladder of a rat model of LOH [37]. Furthermore, in a cryptorchidism-induced rat model, KH-204 treatment alleviated elevations in the levels of heat shock protein and germ cell apoptosis by reducing oxidative stress [38]. In our study, KH-204 treatment prevented the decrease in the expression of eNOS and nNOS and the ratio of muscle/collagen in corpus cavernosum induced by HC, strongly indicating that KH-204 had a preventive effect on HC-associated vascular endothelium and corpora tissue impairment. In particular, decreased 8-OHdG, a predominant marker of oxidative damage to DNA, seemed to indicate that the antioxidant activity of KH-204 prevented oxidative injury under oxidative stress conditions.

Currently, a phosphodiesterase type5 inhibitor (PDE5i) is widely used for treatment of ED. PDE5i acts to block the degradative action of cGMP-specific phosphodiesterase type5 on cGMP in the smooth muscle cells lining the blood vessels supplying the corpus cavernosum of the penis. The most common cause of ED is thought be related to vascular abnormalities caused by endothelial and erectile tissue dysfunction [3, 4], and we demonstrated that the antioxidant effect of KH-204 led to improved erectile function by reducing endothelial and corporal tissue dysfunction from oxidative stress. Therefore, we suggest that KH-204, unlike PED5i, has the potential to therapeutically improve ED by correcting the fundamental cause of ED.

In this study, the group treated with KH204 showed decreased TC, LDL/VLDL-C, and TG and increased HDL-C levels compared to the HFC group. We did not examine, however, why the lipid profile was improved by KH-204 treatment. Although some reports have described the antiobesity and hypolipidemic effects of *Lycium chinense* Miller and *Rubus coreanus* Miquel [24, 25], there is little scholarly evidence available that would explain the improvement in lipid profile seen in our study. Thus, further study should be conducted to elucidate the hypolipidemic mechanisms of this formula.

## Conclusions

Administration of the herbal formula KH-204 to a rat model of HC-induced ED ameliorated lipid profiles and erectile function parameters, including peak ICP and ICP/MAP ratio, expression of eNOS and nNOS, the muscle/collagen ratio, and the level of 8-OHdG in the corpus cavernosum. We believe that the antioxidant properties and hypolipidemic effect of KH-204 make it an effective treatment option for ED, as it can correct a fundamental cause of ED.

## Abbreviations

ANOVA: Analysis of variance
BCA: Bicinchoninic acid
cGMP: Cyclic guanosine monophosphate
DAPI: 4,6-diamidino-2-phenylindole
ED: Erectile dysfunction
ELISA: Enzyme linked immunosorbent assay
eNOS: Endothelial nitric oxide synthase
HC: Hypercholesterolaemia
HDL-C: High-density lipoprotein cholesterol
HFC: High-fat and cholesterol diet
HPLC: High-performance liquid chromatography
ICP: Intracavernous pressure
LDL: Low-density lipoprotein
LOH: Late-onset hypogonadism
MAP: Mean arterial pressure
NADPH: Nicotinamide adenine dinucleotide phosphate
NIH: National Institutes of Health
nNOS: Neuronal nitric oxide synthase
NO: Nitric oxide
PED5i: Phosphodiesterase type5 inhibitor
PMSF: Phenylmethanesulphonyl fluoride
SDS-PAGE: Sodium dodecyl sulphate polyacrylamide gel electrophoresis
TC: Total cholesterol
VLDL: Very low-density lipoprotein
